# Expression of *LhFT1*, the Flowering Inducer of Asiatic Hybrid Lily, in the Bulb Scales

**DOI:** 10.3389/fpls.2020.570915

**Published:** 2020-11-09

**Authors:** Kana Kurokawa, Junya Kobayashi, Keiichirou Nemoto, Akira Nozawa, Tatsuya Sawasaki, Takashi Nakatsuka, Masumi Yamagishi

**Affiliations:** ^1^Graduate School of Integrated Science and Technology, Shizuoka University, Shizuoka, Japan; ^2^Iwate Biotechnology Research Center, Kitakami, Japan; ^3^Proteo-Science Center, Ehime University, Matsuyama, Japan; ^4^Faculty of Agriculture, Shizuoka University, Shizuoka, Japan; ^5^College of Agriculture, Academic Institute, Shizuoka University, Shizuoka, Japan; ^6^Research Faculty of Agriculture, Hokkaido University, Sapporo, Japan

**Keywords:** alternative splicing, cold exposure, flower initiation, *FLOWERING LOCUS T* like genes, geophytes, *Lilium* sp., ornamental plants

## Abstract

Asiatic hybrid lily leaves emerge from their bulbs in spring, after cold exposure in winter, and the plant then blooms in early summer. We identified four *FLOWERING LOCUS T* (*FT*)-like genes, *LhFT1*, *LhFT4*, *LhFT6*, and *LhFT8*, from an Asiatic hybrid lily. Floral bud differentiation initiated within bulbs before the emergence of leaves. *LhFT* genes were mainly expressed in bulb scales, and hardly in leaves, in which the *FT-like* genes of many plants are expressed in response to environmental signals. *LhFT1* was expressed in bulb scales after vernalization and was correlated to flower bud initiation in two cultivars with different flowering behaviors. *LhFT8* was upregulated in bulb scales after cold exposure and three alternative splicing variants with a nonsense codon were simultaneously expressed. *LhFT6* was upregulated in bulb scales after flower initiation, whereas *LhFT4* was expressed constantly in all organs. *LhFT1* overexpression complemented the late-flowering phenotype of *Arabidopsis ft-10*, whereas that of *LhFT8* did so partly. *LhFT4* and *LhFT6* overexpression could not complement. Yeast two-hybrid and *in vitro* analyses showed that the LhFT1 protein interacted with the LhFD protein. LhFT6 and LhFT8 proteins also interacted with LhFD, as observed in AlphaScreen assay. Based on these results, we revealed that *LhFT1* acts as a floral activator during floral bud initiation in Asiatic hybrid lilies. However, the biological functions of *LhFT4*, *LhFT6*, and *LhFT8* remain unclear.

## Introduction

The genus *Lilium* consists of approximately 100 species that are distributed throughout the cold and temperate regions of the Northern Hemisphere and are classified into seven sections (van Tuyl et al., 2018). Lilies are important ornamental plants that include three main distinctive hybrid groups, i.e., Easter lilies, Asiatic hybrid lilies, and Oriental hybrid lilies (Dole and Wilkins, 2005). Asiatic hybrid lilies are derived from interspecific hybridization among *L. dauricum*, *L. maculatum*, *L. lacifolium*, etc., which are species that belong to the sections Sinomartagon and Daurolirion (Marasek-Ciolakowska et al., 2018). Moreover, these plants are characterized by an upward-facing flower and little or no fragrance (Dole and Wilkins, 2005). The color of their flowers is often uniform or with a contrasting perianth segment tips and/or throat, and they exhibit a wide variety of flower colors in different shades, from white to red and yellow (Yamagishi, 2013). Commercial Asiatic hybrid lily cultivars are usually propagated by bulbs, rather than seeds (Beattie and White, 1993).

The typical life cycle of Asiatic hybrid lilies starts with bulb planting in the autumn (October–November), followed by the exposure of bulbs to the low temperatures of winter, which in necessary for flower initiation. Most Asiatic hybrid lilies are vernalized at 1°C–2°C for at least 6 weeks (Nau, 2011). The bulbs sprout in spring and flower in late spring to early summer (Okubo and Sochacki, 2012). Based on the timing of flower bud initiation, 85 Asiatic hybrid cultivars were classified into two types (Ohkawa et al., 1990). In the majority of cultivars (69%), flower bud differentiation starts and is completed after shoot emergence. Conversely, in the remaining cultivars (31%), flower bud initiation commences inside the bulb. With a few exceptions, the former type of Asiatic hybrid lily cultivars flower later than do the latter (Ohkawa et al., 1990). Moreover, no relationship has been identified between shoot growth and flower bud initiation. In *L. longiflorum*, cold exposure is not an obligatory prerequisite for flowering, as an alternative flowering pathway can bypass vernalization in small bulbs (Lazare and Zaccai, 2016). Recently, the levels of glycerol in *L. longiflorum* bulbs was found to be associated with a delay in sprouting and flowering time and a reduction in abortion rate (Lazare et al., 2019).

The floral integrator *FLOWERING LOCUS T* (*FT*) is a key regulator of flowering time in *Arabidopsis* (Corbesier et al., 2007). The FT protein is induced under the flowering-inducive long-day photoperiod in leaves, and is then transported via the phloem to the shoot apical meristem (SAM), where it interacts with the bZIP transcription factor FD (Abe et al., 2005; Corbesier et al., 2007; Tamaki et al., 2007). This protein complex is assumed to comprise two FT monomers and two FD bZIP transcription factors, as well as a dimeric 14–3–3 protein, which acts to bridge the FT–FD interaction (Taoka et al., 2011). The florigen activation complex leads to the direct activation of the floral meristem identity genes, such as *APETALA1* (*AP1*) and *FRUITFULL* (*FUL*) (Taoka et al., 2011). *FT* is a member of the phosphatidyl ethanolamine-binding protein (*PEBP*) gene family (Danilevskaya et al., 2008). *Arabidopsis* carries another *PEBP* gene, *TERMINAL FLOWER 1* (*TFL1*), which determines inflorescence development and suppresses flowering (Bradley et al., 1997). Several amino acids are important for the specific and unique function of FT and TFL1 in *Arabidopsis* (Hanzawa et al., 2005; Ahn et al., 2006). Floral activators such as FT contain a tyrosine (Y) at position 85, whereas floral repressors contain a histidine (H) at the analogous position 88 (Hanzawa et al., 2005). The amino acid residues at position 140 can also affect the function of the protein. In the FT activator, a glutamic acid (Q) is present at position 140, whereas an aspartate (D) is positioned at the same analogous position in the repressors (Ahn et al., 2006). In sugar beets (*Beta vulgaris*), *BvFT2* is the functional *FT* ortholog, while *BvFT1* is a flowering suppressor, despite being in the FT subfamily (Pin et al., 2010). Chrysanthemums (*Chrysanthemum morifolium*) are categorized as absolute short-day plants. In addition, *CsFTL3* encodes a florigen that is induced under short photoperiod conditions in chrysanthemum plants, whereas *CsAFT* encodes an anti-florigen that acts systemically to inhibit flowering under long-day photoperiod conditions and plays a predominant role in the obligate photoperiodic response (Oda et al., 2012; Higuchi et al., 2013; Nakano et al., 2019).

In addition to functioning as activators or repressors of flowering, members of the PEBP family are also involved in a variety of other processes (Wickland and Hanzawa, 2015), such as tuberization (Navarro et al., 2011), bulb formation (Lee et al., 2013), stomatal opening (Kinoshita et al., 2011), and photoperiodic control of seasonal growth in trees (Bohlenius et al., 2006; Hsu et al., 2006, 2011). In *Arabidopsis*, *FT* genes are involved in the regulation of H^++^-ATPase by blue light in stomatal guard cells, resulting in the regulation of stomatal opening by *FT* (Kinoshita et al., 2011). The tuberization of potatoes (*Solanum tuberosum*) under a short-day photoperiod is controlled by a homolog of *FT*, *StSP6A* (Navarro et al., 2011). Interestingly, an *StSP6A*-specific small RNA is induced by elevated temperatures and suppresses the tuberization of potatoes (Lehretz et al., 2019). Four *FT*-like genes (*AcFT1*, *AcFT2*, *AcFT4*, and *AcFT6*) have been identified in onions (*Allium cepa*) (Lee et al., 2013). *AcFT2* is expressed during vegetative growth and likely regulates growth cessation and bud set, which promotes flowering. *AcFT4* functions as an inhibitor of bulbing, whereas *AcFT1* as a promotor of bulbing. Moreover, *AcTFL1* is highly expressed during bulbing and inflorescence development in onions (Dalvi et al., 2019). The expression levels of *AcTFL1* within the bulb are lowest in the outmost layers and highest in the innermost layers.

Some species of the genus *Lilium* have been studied regarding the genes that control flowering and vernalization. An RNA-seq analysis of molecules involved in the vernalization response in the Oriental hybrid lily “Sorbonne” identified two vernalization genes, *SHORT VEGETATIVE PHASE* (*LoSVP*) and *VERNALIZATION 1* (*LoVRN1*), as well as the floral transition key gene *SUPPRESSOR OF OVEREXPRESSION OF CONSTANS 1* (*LoSOC1*) (Liu et al., 2014; Li et al., 2016). Similarly, *L. lancifolium* (which is a breeding material for Asiatic hybrid lilies), the Asiatic hybrid lily “Tiny ghost,” and *L. longiflorum* “White Heaven” were also subjected to transcriptome profiling during the vernalization process by RNA-seq, which led to the identification of several cold signal transduction genes (Huang et al., 2014; Wang et al., 2014; Hamo et al., 2015; Villacorta-Martin et al., 2015). *L. × formolongi*, which is a lily hybrid between *L. formosanum* and *L. longiflorum*, flowers within 1 year of sowing. *CO-LIKE* (*COL*), *FT*, *TREHALOSE-6-PHOSPHATE SYNTHASE* (*TPS*), *SQUAMOSA PROMOTER-BINDING PROTEIN-LIKE* (*SPL*) homologs may play significant roles in the flowering-induction and transition process of *L. × formolongi* (Li et al., 2017). Thus, most flowering studies of the genus *Lilium* were based on integrated expression analyses and the detailed function of *FT*-like genes remains unclear. Conversely, the *LlFT* gene from *L. longiflorum* is upregulated by cold exposure, and the overexpression in *Arabidopsis* and lily plants leads to an early-flowering phenotype (Leeggangers et al., 2018). Therefore, *LlFT* may be involved in the vernalization response of lily and may be able to replace cold exposure.

Several studies of *FT*-like genes have been reported in various monocot horticulture plants, with the exception of lily. Tulips (*Tulipa gesneriana*) carry three *FT*-like genes, *TgFT1*, *TgFT2*, and *TgFT3* (Leeggangers et al., 2017, 2018). *TgFT2* is considered to act as a flowering inducer, as it is involved in floral induction in tulips, whereas *TgFT3* is assumed to have a bulb-specific function. In Chinese narcissuses (*Narcissus tazetta*), which are plants that exhibit summer dormancy, high temperatures are necessary for release from dormancy (Li et al., 2013; Noy-Porat et al., 2013). Under high temperature conditions (25°C–30°C) in the dark, *NtFT* expression occurred simultaneously with floral induction in the bulb meristems of Chinese narcissus plants, indicating that floral induction is affected by high temperature, but not by photoperiod or vernalization (Li et al., 2013; Noy-Porat et al., 2013).

In this study, we attempted to isolate and characterize *FT*-like genes in ornamental Asiatic hybrid lily.

## Materials and Methods

### Plant Materials

**FIGURE 1:**
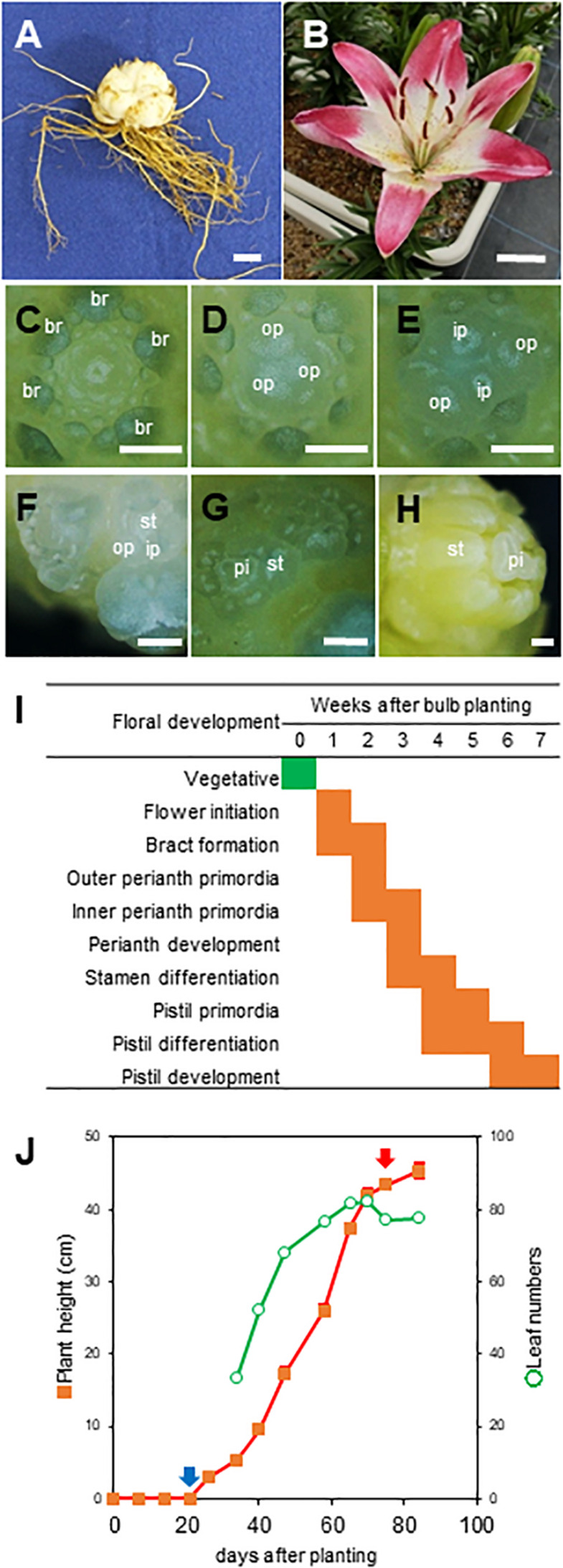
Vegetative and reproductive development stages in the Asiatic hybrid lily ‘Lollypop.’ **(A)** Bulbs before chilling exposure. Bar, 2 cm. **(B)** Anthesis. Bar, 2 cm. (**C–H)** Definition of the floral development stages of the SAM. Bar, 500 μm. br, bract; op, outer perianth; ip, inner perianth; st, stamen; pi, pistil. **(C)** Vegetative SAM. **(D)** Flower initiation, rounded SAM. **(E)** Bract formation. **(F)** Formation of outer and inner perianthes primordia. **(G)** Formation of pistil primordia. **(H)** Pistil development. **(I)** Flower bud transition after planting. Flower developmental stages are defined in “Materials and Methods.” We observed three independent SAMs each week. **(J)** Alteration of plant height and leaf number after planting. The blue arrow indicates leaves emerged from the soil at three weeks after planting. The red arrow indicates ‘Lollypop’ blooming at 75 days after planting.

The bulbs (circumference, 16 cm) of the Asiatic hybrid lily ‘Lollypop’ [original name, ‘Holebibi’ (Matthews, 2007)] were purchased from the Niigata Flower Bulb Growers Cooperative Association (Niigata, Japan) in September 2016 and 2017 (Figures 1A,B). The bulbs of *L. leichtlinii* ‘Hakugin’ which is one of the breeding materials of this Asiatic hybrid lily, were also harvested in August 2015 from a field of Hokkaido University (Sapporo, Japan) and were stored at 20°C until use. Cold treatment of bulbs was performed in peat moss in the dark at 4°C for 4 months. In early March, all bulbs were planted at a density of 12 bulbs per 16 L planter, which was filled with a mix of akadama and leaf mold (2:1) containing 2.5 g⋅L^–1^ Magamp K (Hyponex Japan, Osaka, Japan). The plants were grown in a field of Shizuoka University (Shizuoka, Japan) under natural conditions.

During cold treatment and after planting, scales, SAMs, and leaves were sampled. The second and third outer scales and SAMs containing the basal plate were collected from bulbs, whereas SAMs with a height of approximately 1 cm were collected from sprouted plants. The mature leaves were also collected from sprouted plants.

### Observation of Floral Development

**FIGURE 2:**
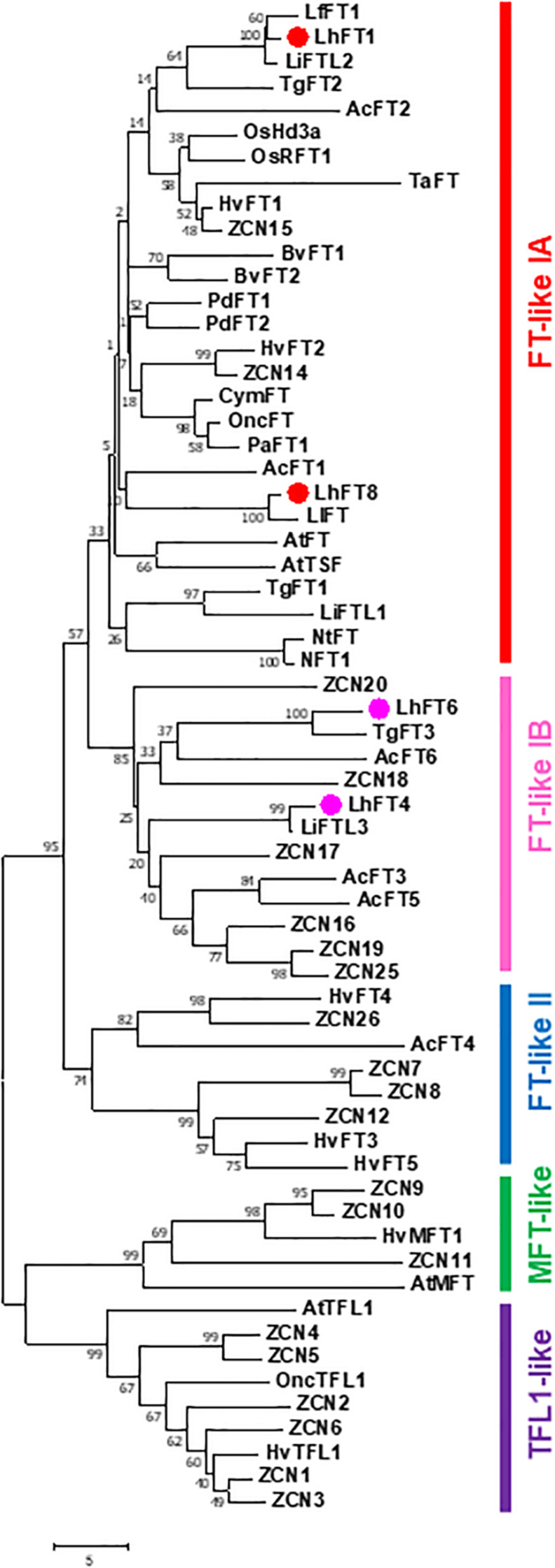
Phylogenetic tree of FT-like protein sequences from different monocot plants. The phylogenetic tree was constructed using the neighbor-joining method. Bootstrap values from 1,000 replicates were used to assess the robustness of the tree. The scale indicates the average number of substitutions per site. The *FT*-like gene names and GenBank accession numbers were as follows: *Allium cepa AcFT1* (AG081838.1), *AcFT2* (AGZ20208.1), *AcFT3* (AGZ20209.1), *AcFT4* (AGZ20210.1), *AcFT5* (AGZ20211.1), and *AcFT6* (AGZ20212.1); *Arabidopsis thaliana AtFT* (NP_176726.1), *AtMFT* (NP_173250.1), *AtTFL1* (NP_196004.1), and *AtTSF* (NP_193770.1); *Cymbidium goeringii CymFT* (ADI58462.1); *Hordeum vulgare HvFT1* (AAZ38709.1), *HvFT2* (ABB99414.1), *HvFT3* (ABD75336.2), *HvFT4* (ABD75337.1), *HvFT5* (ABM26903.1), *HvMFT1* (BAH24198.1), and *HvTFL1* (ANO81637.1); *Lilium* hybrid *LfFT1* (AIF76295.1), *LhFT1*, *LhFT4*, *LhFT6*, and *LhFT8* (this study); *L. longiflorum FlFT* (ATU06687.1); *Lilium* sp. *LiFTL1* (ATU06689.1), *LiFTL2* (ATU06688.1), and *LiFTL3* (ATU06690.1); *Narcissus tazetta NtFT* (AFS50164.1); *Oncidium* hybrid *OncFT* (AIG6303.1) and *OncTFL1* (AIU44253.1); *Oryza sativa OsHd3a* (XP_01641951.1) and *OsRFT1* (XP_015642519.1); *Phalaenopsis aphrodite PaFT1* (AJF93423.1); *Triticum aestivum TaFT* (ACA25437.1); *Tulipa gesneriana TgFT1* (ATU06684.1), *TgFT2* (ATU006685.1), and *TgFT3* (ATU006686.1); *Zea mays ZCN1* (ONM42318.1), *ZCN2* (NP_00110624.1), *ZCN3* (NP_001106242.1), *ZCN4* (NP_001106243.1), *ZCN5* (NP001106244.1), *ZCN6* (NP_001106245.1), *ZCN7* (NP_001106246.1), *ZCN8* (NP_001106247.1), *ZCN9* (ABW96232.1), *ZCN10* (NP_00106249.1), *ZCN11* (AQK83984.1), *ZCN12* (NP_001106250.1), *ZCN14* (NP_001106251.1), *ZCN15* (NP_001106252.1), *ZCN16* (NP_001106253.1), *ZCN17* (NP_001106254.1), *ZCN18* (NP_001354358.1), *ZCN19* (NP_001106256.1), *ZCN20* (NP_001296779.1), *ZCN25* (NP_001106257.1), and *ZCN26* (NP_001106265.1).

**FIGURE 3:**
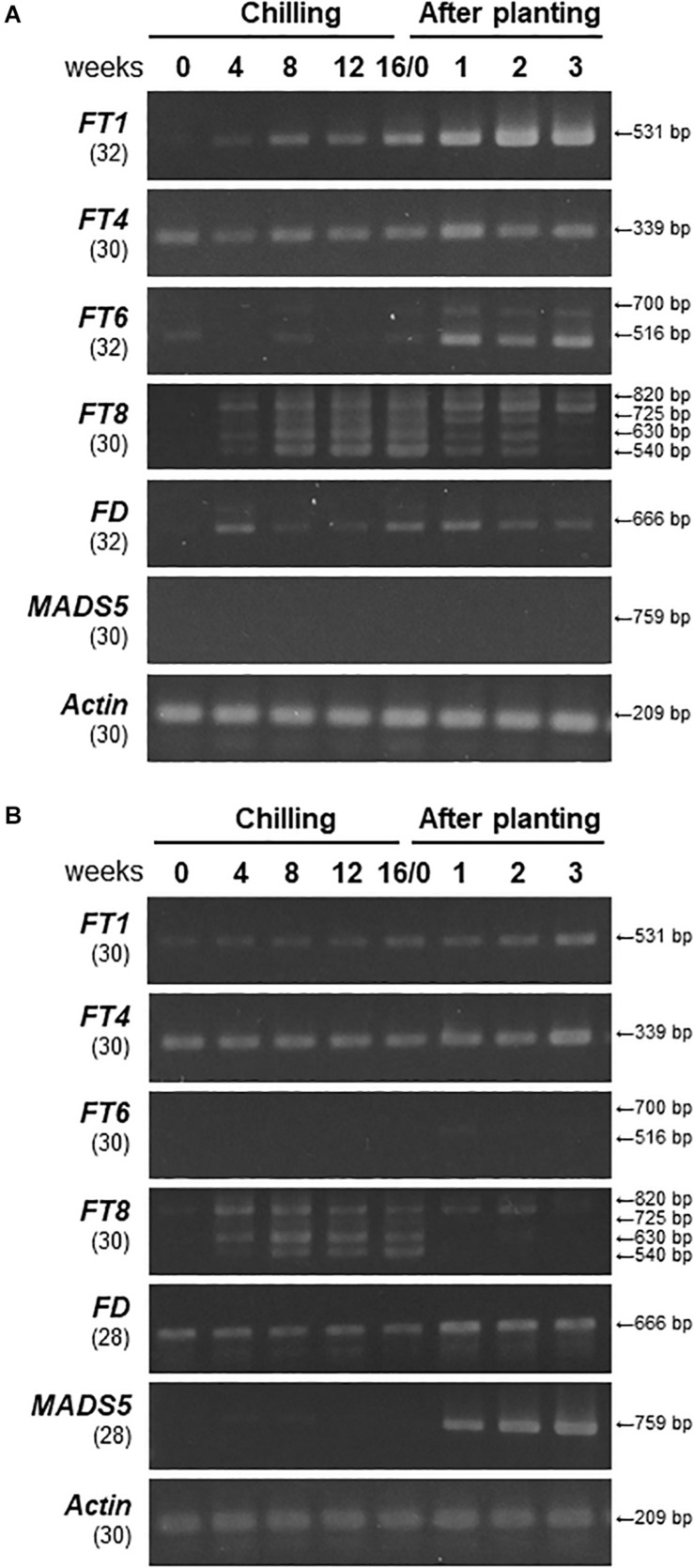
Expression patterns of *LhFT* and flowering-related genes in ‘Lollypop’ bulbs during chilling and planting. **(A)** Semiquantitative RT-PCR analysis of *LhFT, LhFD*, *LhMADS5*, and *LhACT* genes using scales (**A**) and SAMs **(B)** of ‘Lollypop’ blubs. Scale and SAM samples were collected from bulbs exposed to chilling for 0, 4, 8, 12, and 16 weeks, and from bulbs grown for 1, 2, and 3 weeks after chilling for 16 weeks. The gene names and number of cycles are indicated to the left of the panel. The amplified fragment lengths are indicated to the right of the panels. The 516-bp fragment of *LhFT6* corresponds to the normal transcript, whereas the 700-bp fragment corresponds to the alternative transcript. The 540-bp fragment of *LhFT8* corresponds to normal transcript, whereas the 630-bp, 725-bp, and 820-bp fragments correspond to alternative transcripts.

**FIGURE 4:**
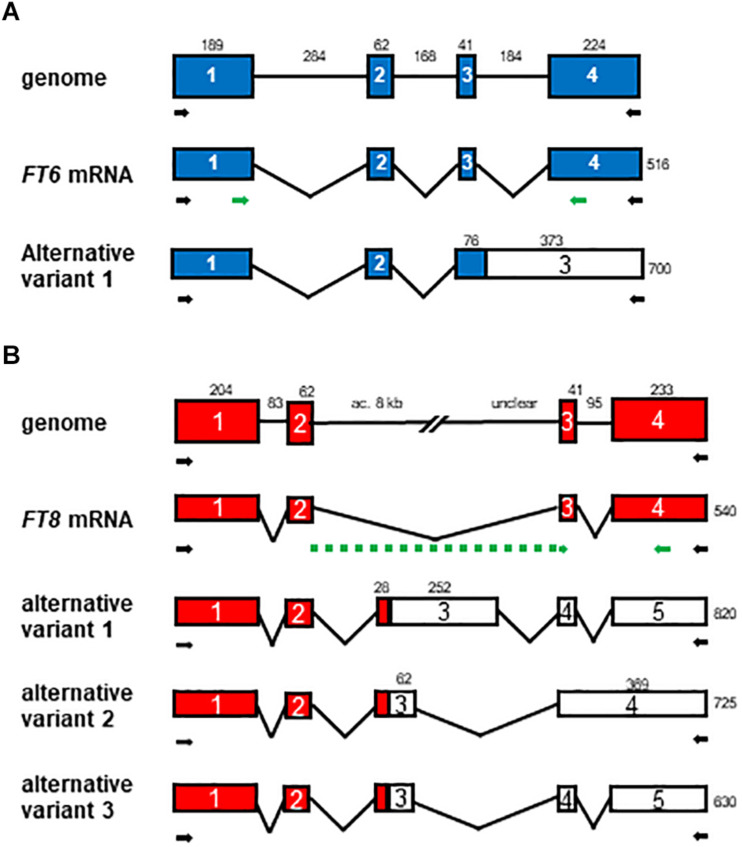
Structure of the genome and mRNA variants of *LhFT6* **(A)** and *LhFT8* **(B)**. The boxes and lines indicate exons and intron, respectively. Filled and empty boxes indicate coding and non-coding regions, respectively. The numbers within the boxes correspond to the number of exons, whereas the numbers outside the boxes indicate the length (in base pairs). We were not able to obtain the complete sequences of the second intron of *LhFT8* because it was too long to allow amplification and sequence analysis. Black and green arrows indicate primer position in RT-PCR and RT-qPCR analyses.

Floral differentiation of SAM was observed in at least three independent samples for each point using a stereomicroscope. Floral developmental stage was defined as follows: (1) Vegetative (Figure 1C); (2) flower initiation (Figure 1D); (3) bract formation (Figure 1E); (4) outer perianth primordia (Figure 1F): (5) inner perianth primordia, (6) perianth development; (7) stamen differentiation; (8) pistil primordia (Figure 1G); (9) pistil differentiation; and (10) pistil development (Figure 1H; Fukai and Goi, 2001).

### Isolation of *FT* Orthologs From an Asiatic Hybrid Lily

The deposited transcriptome data (ERR578452 to ERR578471) of *L. longiflorum* were assembled using the trinity program of the DDBJ read annotation pipeline (Nagasaki et al., 2013); 300,375 contigs were obtained. *Lilium FT/TFL1* orthologs were identified in these contigs by the blast program using *FT*/*TFL1* orthologs from several plant species as queries. Several primers based on the nucleotide sequences of nine *FT*/*TFL1* candidate genes from *L. longiflorum* were designed to amplify *FT*/*TFL1* and flowering-related gene orthologs from an Asiatic hybrid lily, as shown in Supplementary Table 1.

Shoot apical meristems, scales, and leaves of *L. leichtlinii* ‘Hakugin’ were sampled at several developmental stages. Total RNA was isolated from each sample using the Fruit mate for RNA purification (Takara Bio, Shiga, Japan) and RNAiso Plus (Takara Bio). cDNAs were synthesized using a PrimeScript II 1st strand cDNA synthesis kit (Takara Bio). Using the primers that were designed as described above, we amplified putative *FT*/*TFL1* orthologs using the *L. leichtlinii* ‘Hakugin’ cDNA. The reaction mixture (25 μL) consisted of 1× *Ex Taq* buffer, 200 μM dNTPs, 0.2 μM each primer, 0.25 U of *Ex Taq* polymerase (Takara Bio) and 1 μL of template cDNA. The thermal cycler program was set as follows: 94°C for 2 min; followed by 35 cycles of 94°C for 20 s, 40°C–55°C for 40 s, and 72°C for 1 min; and a final step at 72°C for 10 min. The amplified fragments were subcloned into the pGEM-Teasy vector system (Promega, Madison, WI, United States). The sequences of all constructs were confirmed by DNA sequencing (Fasmac, Kanagawa, Japan).

Based on the partial sequences obtained for each *FT/TFL1* fragment, we used the rapid amplification of cDNA ends (RACE) technology to obtain full-length cDNA sequences with the SMARter RACE 5′/3′ kit (Takara Bio). The open reading frame (ORF) sequences were amplified using *Ex Taq* polymerase, and the primer sets are listed in Supplementary Table 1. The thermal cycler program was set as follows: 94°C for 2 min; followed by 35 cycles of 94°C for 20 s, 60°C for 40 s, and 72°C for 2 min; and a final step at 72°C for 10 min. The amplified fragments were cloned and sequenced as described above. A phylogenetic tree of FT-like protein was constructed using ClustalW with neighbor-joining algorithm and visualized using MEGA ver. 7 software (Kumar et al., 2016).

### Expression Analysis

Total RNA was isolated from the SAMs, scales, and leaves of each treated plant. cDNA was synthesized from total RNAs, as described above. For the RT-PCR analyses, a reaction mixture (50 μL) consisted of 1 × *Ex Taq* buffer, 200 μM of dNTPs, 0.4 μM of each primer, 0.25 U of *Ex Taq* polymerase Hot Start version (Takara Bio), and 1 μL of template cDNA. The PCR cycling conditions were as follows: 2 min at 94°C; 26–34 cycles for 20 s at 95°C, 40 s at 55°C, and 1 min at 72°C; and final extension for 10 min at 72°C. The sequences of the primers are listed in Supplementary Table 2. The PCR products were separated on 1.5% agarose gels in TAE buffer and stained with ethidium bromide.

Reverse transcription-quantitative PCR (RT-qPCR) analyses used a Thermal Cycler Dice Real-Time System (TP850, Takara Bio), according to MIQE guidelines (Bustin et al., 2009). Briefly, the reaction mixture (10 μL) consisted of 1× KAPA SYBR Fast qPCR Master Mix (KAPA Biosystems, Wilmington, MA, United States), 0.2 μM of each primer, and 1 μL of template cDNA. Cycling conditions were as follows: 95°C for 20 s; followed by 40 cycles of 95°C for 1 s and 60°C for 20 s. The sequences of the primers used in this study are listed in Supplementary Table 2. The specificity of amplification was checked by the addition of a dissociation analysis step after the cycle reactions. Data were analyzed by second derivative maximum methods using Thermal Cycler Dice Real-Time System II software version 5.00 (Takara Bio). Transcript levels were calculated relative to the actin-encoding *LhACT* gene (AB438963) used as a reference (Yamagishi et al., 2014; Suzuki et al., 2016). In a preliminary experiment, similar amplification rates of *LhACT* were detected among scale, SAM, and leaf samples of ‘Lollypop’ at different developmental stages (Supplementary Figure 1). The RT-qPCR analyses used six biological replicates.

### Yeast Two-Hybrid Analysis Between LhFT Proteins and LhFD

To investigate whether the LhFT proteins interact with the LhFD protein, we employed a yeast two-hybrid analysis using the Matchmaker Two-Hybrid System 3 (Clontech, Takara Bio, Shiga, Japan) as described previously (Nakatsuka et al., 2019). The coding regions of *LhFT1*, *LhFT4*, *LhFT6*, *LhFT8*, and *LhFD* were cloned into either pGAD-T7 (GAL4 activation domain) or pGBK-T7 (GAL4 DNA-binding domain) vectors. All the constructs were transformed into *Saccharomyces cerevisiae* AH109 (Clontech). Transformed yeast cells were grown on SD selective medium without leucine (−Leu) and tryptophan (−Trp) at 30°C for 3 days. A survival test was performed for each transformed yeast culture using selective quadruple-dropout medium (−Leu, −Trp, histidine [−His], and adenine [−Ade]) supplemented with 15 mM of 3-amino-1,2,4-triazole (3-AT) at 30°C for 3 days.

### AlphaScreen-Based *in vitro* Protein–Protein Interaction Assay

The ORFs of *LhFTs*, *LhFD*, *AtFT*, and *AtFD* were modified with two-step PCR using gene-specific primer pairs with S1 or T1 linker sequences for the first step and primers attB1-S1 and attB2-T1 for the second step. The DNA fragments were cloned into pDONR221 vector using the gateway cloning system (Thermo Fisher Scientific). These expression vectors were generated using LR clonase recombination with pEU-E01-GW-AGIA (Yano et al., 2016) or pEU-E01-GW-bls (Iwasaki et al., 2016) vector for cell-free protein synthesis or transient expression vectors. All primer sequences are listed in Supplementary Table 3.

*In vitro* transcription and wheat cell-free protein synthesis were performed using the WEPRO1240 expression kit (Cell-Free Sciences, Matsuyama, Japan) according to the manufacturer’s instructions. *In vitro* biotin labeling of recombinant protein was performed as previously described (Sawasaki et al., 2008).

AlphaScreen-based *in vitro* protein–protein interaction assay was performed as previously described with slight modifications (Nemoto et al., 2017). The assay was performed using a reaction mixture of 15 μL containing AlphaScreen buffer [100 mM Tris–HCl (pH 8.0), 0.1% Tween20, 1 mg⋅mL^–1^ BSA], 1 μL of C-terminal biotinylated LhFD, and 1 μL of C-terminal AGIA-tagged LhFTs in a 384-well Optiplate (PerkinElmer, Waltham, MA, United States). After incubation at 25°C for 1 h, 10 μL of detection mixture containing AlphaScreen buffer, 1 μg⋅mL^–1^ anti-AGIA antibody (Yano et al., 2016), 0.1 μL of streptavidin-coated donor beads (PerkinElmer), and 0.1 μL of protein A-coated acceptor beads (PerkinElmer) were added to each well of the 384-well Optiplate, followed by incubation at 25°C for 1 h. Luminescence was analyzed using the AlphaScreen detection program. All data represent the average of three independent experiments, and the background was controlled using a biotinylated dihydrofolate reductase (DHFR) from *E. coli*.

### Complement Expression of *LhFT* Genes in the *Arabidopsis ft-10* Mutant

The ORFs of *LhFTs* were located between the cauliflower mosaic virus (CaMV*) 35S* promoter and the *Arabidopsis* heat-shock protein terminator (HT) of a binary vector, pShyg-35SproGUS-HT the hygromycin-resistance gene was modified from the pSMAB704 backbone vector (Igasaki et al., 2002). These constructs were then transformed into the *Agrobacterium tumefaciens* EHA101 strain. *Arabidopsis ft-10* mutant was transformed using the floral dip method, as described (Clough and Bent, 1998). Positive transformants were selected on germination medium supplemented with 30 mg⋅L^–1^ hygromycin, with T_2_ seeds being obtained after self-pollination. Transgenic plants were grown at 22°C under 16-h day florescence light. Homozygotic T_2_ lines of each transgenic plant were used to investigate flowering time.

The total RNA was isolated from the mature leaves of 3-week-old seedling in Col-1 and T_2_ line, as described above. The expression levels of transgene *LhFTs* and *AtACT2* as internal standard were investigated by RT-PCR analysis, as described above.

### Statistical Analysis

Data are presented as the mean ± SE. Statistical comparisons were carried out using Tukey–Kramer and Student’s *t*-tests.

## Results

### Growth Behavior of the Asiatic Hybrid Lily ‘Lollypop’ and *L. leichtlinii* ‘Hakugin’

Before the investigation of the temporal and spatial expression of *LhFT* genes in pre-vernalization and during planting, we examined the floral development stages of the SAM of bulbs in each phase (Figure 1I). The SAM of bulbs that were pre-chilled over 4 months was kept in the vegetative phase. One week after planting on soil, the SAM of bulbs exhibited either a swollen round apex or produced a bract. Two weeks after planting, SAMs displayed perianth differentiation. Therefore, the SAM of bulbs of ‘Lollypop’ was assumed to engage in flower bud initiation at ∼2 weeks after planting. Three weeks after planting, leaves emerged from the soil, and flower buds were observed 8 weeks after planting (Figure 1J). The increase in the number of developed leaves stopped 82.3 ± 1.7 leaves at 10 weeks after planting, and flowers bloomed completely at 75 days after planting, resulting in a plant height of 45.3 ± 1.0 cm (Figure 1J).

*Lilium lacifolium* ‘Hakugin’ was observed to be swollen around the apex at 7 weeks after planting in the soil, and it bloomed at 16 weeks after planting. Both floral initiation and blooming of ‘Hakugin’ was 6 weeks later compared to those of Asiatic hybrid ‘Lollypop.’

### Isolation of *LhFT* Orthologs

We obtained four *FT*-like genes, which were termed *LhFT1* (accession number, LC544113), *LhFT4* (LC544114), *LhFT6* (LC544115), and *LhFT8* (LC544117), from *L. leichtlinii* ‘Hakugin.’ A phylogenetic analysis showed that they were classified into two subgroups (Figure 2). *LhFT1* and *LhFT8* were classified into the *FT*-like IA subgroup. The deduced amino acid sequence of *LhFT1*, which belonged to the FT-like IA subgroup, exhibited 96.6% and 94.9% identity with that of *LfFT1* from *L.* × *formolongi* (Li et al., 2017) and *TgFT2* from *T. gesneriana* (Leeggangers et al., 2018), respectively. The deduced amino acid sequence of *LhFT8* exhibited 96.6% and 77.5% identity with that of *LlFT* from *L. longiflorum* (Leeggangers et al., 2018) and *PaFT1* of *Phalaenopsis aphrodite* (Jang et al., 2015). *LhFT1* showed 72.2% identity with *LhFT8* based on amino acid sequences (Supplementary Figure 2). Conversely, *LhFT4* and *LhFT6* were classified into the *FT*-like IB subgroup (Figure 2). *LhFT4* exhibited 75.7% identity with *LhFT6* based on amino acid sequences. The deduced amino acid sequence of *LhFT6* showed 86.5% identity with that of *TgFT3* from *T. gesneriana* (Leeggangers et al., 2018) and *AcFT6* from *A. cepa* (Lee et al., 2013), respectively. The deduced amino acid sequence of *LhFT4* correlated well with that of *LiFTL3* (98.1% identity), as reported by Leeggangers et al. (2018). The *LhFT4* mRNA had a length of 1,164 bp, but its maximum ORF was 339 bp, from the 447th to 815th nucleotides, and encoded 112 amino acid residues. Thus, LhFT4 protein was shorter than LiFTL3 protein (181 residues), which resulted from the shortening of its N terminus (Supplementary Figure 2). After isolation of *LhFTs* from *L. leichtlinii* ‘Hakugin,’ we confirmed nucleotide sequences of the corresponding genes in the Asiatic hybrid ‘Lollypop,’ which showed 7 to 10 nucleotide substitutions compared with those of ‘Hakugin.’ Therefore, we designed primers for expression analysis based on the conserved sequences of *LhFTs* in ‘Lollypop’ and ‘Hakugin.’

LhFT1, LhFT6, and LhFT8 proteins contain a tyrosine (Y, FT-type) at positions 87, 89, and 84, respectively, whereas LhFT4 protein contains a histidine (H, TFL1-type) residue at position 17 (Supplementary Figure 2). In segment B motif, LhFT1, LhFT4, and LhFT8 proteins contain a glutamine (Q, FT-type) at positions 139, 72, and 141, respectively, whereas the LhFT6 protein contains a proline (P) residue at position 136 (Supplementary Figure 2). Aspartic acid (D) at position 17 and valine (V) at position 18, which are important for the transport of FT, are conserved in the LhFT1, LhFT6, and LhFT8 proteins. Taken together, these results suggest that LhFT1 and LhFT8 are very similar to FT-like proteins, whereas LhFT4 is similar to TFL1-like proteins. Moreover, LhFT6 did not exhibit several characteristic features of the FT protein but was similar to the TgFT3 protein from tulips.

In addition to the four *FT*-like genes, we also identified one *FD* ortholog, *LhFD* (LC544121). The *LhFD* mRNA had a length of 816 bp and encoded a sequence of 221 amino acids. The phylogenetic tree showed that LhFD was classified into eudicots and non-Poacea monocots subgroup (Supplementary Figure 3). The deduced amino acid sequence of *LhFD* exhibited 34.6% and 41.0% identity with that of *AtFD* from *A. thaliana* (Abe et al., 2005) and *PaFD* from a *P. aphrodite* (Jang et al., 2015), respectively. The deduced amino acid sequences of *LhMADS5* exhibited 95.6% identity with *AP1* ortholog of *L. longiflorum* (ADT78582, Chen et al., 2008).

### Spatial and Temporal Expression Profiles of *FT*-Like Genes in the Asiatic Hybrid Lily ‘Lollypop’ and *L. leichtlinii* ‘Hakugin’

To investigate the expression profiles of *LhFT* genes in ‘Lollypop’ bulbs during the period from chilling exposure of bulbs to leaf emergence from the soil, we also performed a semiquantitative RT-PCR analysis using primer sets that amplified the ORF of each gene. The expression of *LhFT1* in bulb scales increased gradually as chilling exposure progressed and was further increased after planting (Figure 3A). Therefore, it was thought that the activation of *LhFT1* transcription occurred right after bulb planting. Similarly, the expression of *LhFT6* was activated in the scales after planting (Figure 3A). Two *LhFT6* mRNA variants were detected in the bulb scales (Figure 3A), whereas no amplified fragment of this gene was observed in SAMs throughout the chilling and planting periods (Figure 3B). The *LhFT6* genome was composed of four exons and three introns, and its full length (from the initial codon to the stop codon) was 1,152 bp (Figure 4A). The amplified fragment of 700 bp corresponding to the alternative mRNA failed to splice out the third intron of *LhFT6* (Figure 4A). This alternative *LhFT6* variant included a nonsense codon at position 370 from the initial codon, leading to the lack of the C-terminal region containing segment B (Supplementary Figure 2). *LhFT6* was weakly amplified, whereas alternative *LhFT6* variant was strongly amplified (Figure 3A). The activation of the expression of *LhFT1* and *LhFT6* in the bulb scales was correlated with floral initiation from SAMs (Figures 1, 3A). Four amplified fragments were detected in *LhFT8* in both scales and SAM of bulbs from 4 weeks after chilling to 2 weeks after planting (Figures 3A,B). The genome structure of *LhFT8* was composed of four exons and three introns, but its second intron could not be assessed completely using TAIL-PCR and inverted PCR because it was too long (>8 kb). Three longer *LhFT8* mRNA variants that were detected in scales at 12 weeks after chilling exposure were cloned and sequenced. The fragment of 820, 725, and 630 bp corresponding to alternative *LhFT8* variants included 280 bp or 90 bp *de novo* exons within the second intron (Figure 4B) and carried a nonsense codon at position 292 from the initial codon (Supplementary Figure 2). *LhFT8* was observed in both bulb scales and SAMs after 4 weeks of chilling exposure and decreased after planting (Figures 3A,B). An alternative *LhFT8* variant 1 increased in the bulb scales after planting and remained constant thereafter. *LhFT4* was expressed constantly in both scales and SAMs during chilling and planting (Figures 3A,B). Although the expression of *LhFD* was detected in both scales and SAMs, it was stronger in SAMs than it was in scales. The expression of *LhMADS5*, which is a floral identity gene, was detected exclusively in SAMs at planting and 1 week after planting, which implied that floral bud differentiation started in SAMs (Figures 1, 3B).

We also investigated the expression levels of *LhFTs* and *LhMADS5* in bulb scales of *L. leichtlinii* ‘Hakugin’ (Supplementary Figure 4). The expression of *LhFT1* was detected 1 week after planting, which then increased markedly at 4 weeks after planting. The expression profiles of *LhFT6* were similar to those of *LhFT1*. *LhFT8* was detected in alternative splicing variants, and its expression decreased within two weeks of planting. No expression of *LhFT4* was detected in bulb scales. Increased expression of *LhFT1* and *LhFT6* at 4–6 weeks after planting was correlated with flower initiation, which occurred 7 weeks after planting.

### Quantitative Expression Profiles of *FT*-Like Genes in the Asiatic Hybrid Lily ‘Lollypop’

**FIGURE 5:**
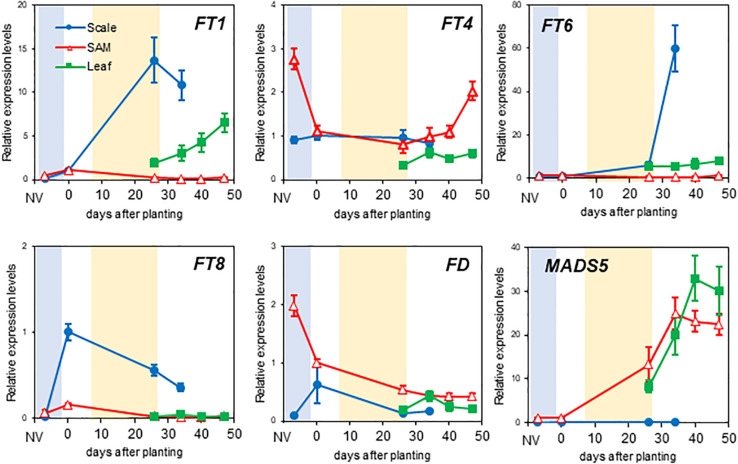
Temporal expression patterns of *LhFT* and flowering-related genes in different organs of ‘Lollypop.’ The quantitative gene expression analysis of *LhFT1*, *LhFT4*, *LhFT6*, *LhFT8*, *LhFD*, and *LhMADS5* in SAM, scales, leaf samples of Asiatic hybrid ‘Lollypop’ was performed. Bulb scales samples were collected from bulbs under different development phase, including non-vernalization (NV) and vernalization (= 0 day after planting), 26, and 34 days after planting. Leaf samples were collected from 26, 34, 40, and 47 days after planting. SAM samples were collected from non-vernalized bulbs and vernalized bulb (0 day) and from shoots at 26, 34, 40, and 47 days after planting. Blue and orange fill boxes indicated chilling exposure period for 4 months and floral initiation period, respectively. The expression levels of *LhFT* and flowering-related genes were investigated by RT-qPCR and normalized using that of *LhACT*. Values are expressed as the mean ± SE (*n* = 6). Temporal expression pattern per each organ is shown in Supplementary Figure 5.

Using RT-qPCR, we investigated the expression profiles of *LhFT* and flowering-related genes using bulb scales, leaves, and SAMs of ‘Lollypop’ (Figure 5). The primer set used for *LhFT6* and *LhFT8* genes in the RT-qPCR analysis did not detect alternative variants (Figure 4). The expression of *LhFT1* was detected mainly in bulb scales, and not in SAMs. The expression levels of *LhFT1* were increased by 13.7-fold in bulb scales at 26 days compared with day 0 after planting, whereas the expression levels of *LhFT1* were weak in just-emerged leaves (Figure 5). The strong expression of *LhFT1* in bulb scales continued until 34 days after planting, after which the mother bulbs disappeared gradually. *LhFT4* was detected at weak but constant levels in bulb scales and leaves during plant development. In the SAM of non-vernalized bulbs and in differentiated floral meristem, the expression levels of *LhFT4* were about 2-fold those detected in other phases. The expression of *LhFT6* was detected exclusively in bulb scales, and was 59.8-fold higher at 34 days compared with day 0 after planting (Figure 5). This peak in *LhFT6* expression was observed 1 week later than that of *LhFT1*. The highest expression level of *LhFT8* was detected in bulb scales right after chilling exposure for 4 months, and was 75.6-fold higher than that of non-vernalized bulbs (Figure 5). Furthermore, the expression levels of *LhFT8* in bulb scales were reduced after planting. In leaves and SAMs, *LhFT8* transcripts were hardly detected throughout the investigation period. *LhFD* exhibited its highest expression levels in SAMs of non-vernalized bulbs. Subsequently, the expression levels of *LhFD* were reduced by 50% after chilling exposure and planting. Modest expression peaks of *LhFD* in bulb scales and leaves were detected right after chilling exposure and at 34 days after planting, respectively. The expression of *LhMADS5*, which is an *AP1* ortholog, was increased in SAMs after planting, and peaked at 34 days after planting. The expression levels of *LhMADS5* were 24.9-fold higher at 34 days compared with day 0 after planting. The expression of *LhMADS5* was also detected in leaves and was increased up to 40 days after planting.

Taken together, these results showed that enhanced expression of *LhFT1* and *LhFT6* was detected in bulb scales 1 week after planting and correlated well with the timing of floral initiation in SAMs. Conversely, *LhFD* was expressed constantly in SAMs throughout the chilling and planting periods, but was also expressed weakly in scales. Finally, *LhFT8* was expressed during chilling exposure.

### Overexpression in the *Arabidopsis ft-10* Mutant

**FIGURE 6:**
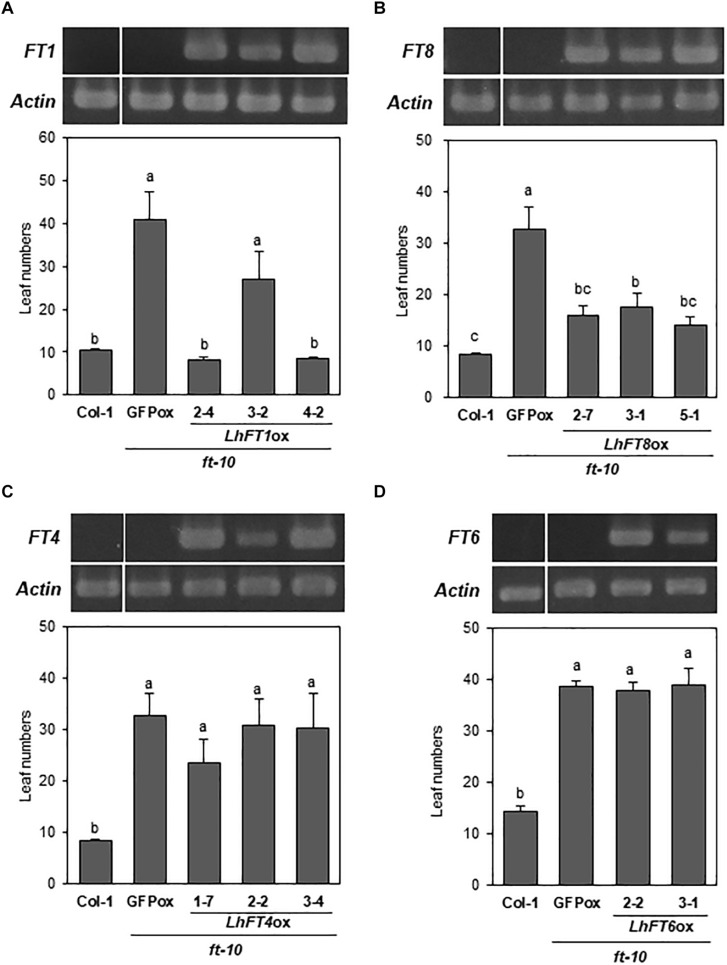
Flowering time phenotypes of the lines overexpressing *LhFT* genes in the *Arabidopsis ft-10* mutant. **(A)** *LhFT1*-overexpressing transgenic plants. **(B)** *LhFT8*-overexpressing transgenic plants. **(C)** *LhFT4*-overexpressing transgenic plants. **(D)** *LhFT6*-overexpressing transgenic plants. All transgenic plants are T_2_ homozygous specimens. Col-1 represents the wild-type. GFP indicates the vector control transgenic plants in the *Arabidopsis ft-10* background. RT-PCR analysis was performed to investigate the expression levels of transgenes and *AtACT2* (internal standard). The number of leaves in transgenic lines overexpressing each *LhFT* gene was investigated in 10 individuals of two or three independent lines during bolting. Values are expressed as the mean ± SE (*n* = 10). The different letters placed above columns are significantly different according to the Tukey–Kramer test (*P* < 0.05).

To evaluate the flowering-induction activity of *LhFT* genes, we produced transgenic plants using the late-flowering *Arabidopsis ft-10* mutant. We selected tree representable T_2_ lines for each construct and investigated their gene expression patterns and phenotypes. In all selected transgenic T_2_ lines, the expression of transgenes was detected (Figure 6). Any alternative splicing variants were not detected in *LhFT6*- and *LhFT8*-overexpressing plants. *LhFT1*-expressing plants nos. 2-1, 3-2, and 4-2 developed 8.1, 27.0, and 8.4 rosette leaves, respectively, during bolting, which was significantly lower compared with the 40.9 rosette leaves observed in *GFP*-expressing *ft-10* plants as a vector control (Figure 6A). The overexpression complemented the *ft-10* mutant, resulting in similar early-flowering time compared with the wild-type Col-1 plants (10.4 rosette leaves). Similarly, *LhFT8*-expressing plants nos. 2-7, 3-1, and 5-1 developed 15.8, 17.6, and 14.1 rosette leaves, respectively (Figure 6B), which was significantly lower than that observed in the *GFP*-expressing plant but higher than that in Col-1 and *LhFT1*-expressing plants nos. 2-4 and 4-2 (Figures 6A,B). Therefore, the overexpression also partially complemented the *ft-10* mutant phenotype. Conversely, *LhFT4* and *LhFT6* overexpression did not complement the *ft-10* mutant (Figures 6C,D). Taken together, these results suggest that *LhFT1* and *LhFT8* are potential inducers of the floral transition in Asiatic hybrid lilies.

### Protein–Protein Interactions Between LhFT Proteins and LhFD

**FIGURE 7:**
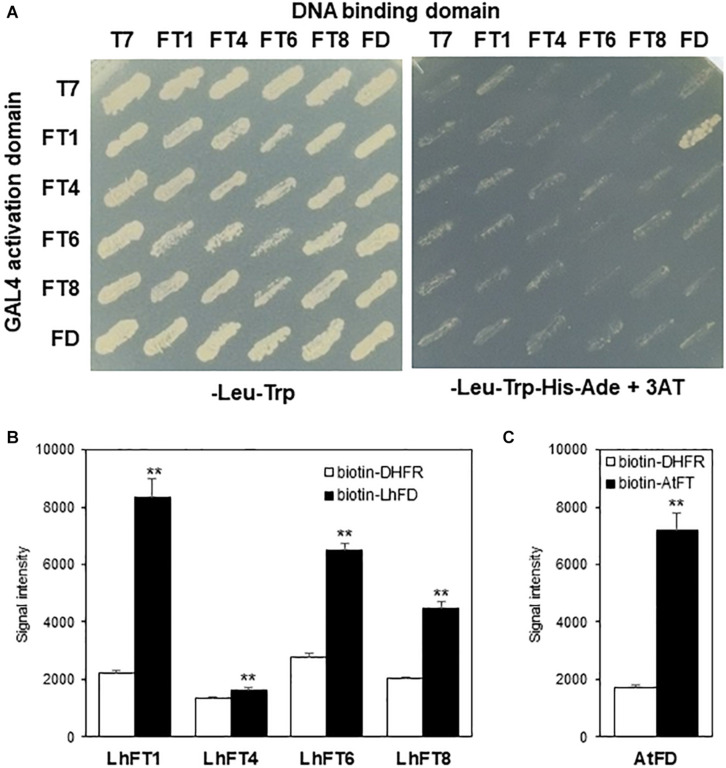
Protein–protein interactions between the LhFT proteins and LhFD. **(A)** The LhFT1, LhFT4, LhFT6, LhFT8, and LhFD proteins were fused to the GAL4 DNA-binding domain (BK) or GAL4 activation domain (AD). pGBKT7 and pGADT7 were used as the negative controls, for bait and prey, respectively. Yeast cells were grown on double-selection medium (−Leu, and −Trp; left) and quadruple-dropout medium (−Leu, −Trp, −His, and −Ade) supplemented with 15 mM 3-AT (right) at 30°C for 3 days. **(B)** *in vitro* protein–protein interaction assay by AlphaScreen. AGIA-tagged LhFTs were incubated with biotinylated LhFD. The interaction intensity between LhFTs and LhFD was analyzed by AlphaScreen Biotinylated dihydrofolate reductase (DHFR), and *E. coli* was used as negative control. Data are mean ± SD. of three independent experiments (*n* = 3). **indicates the significant difference with student’s *t*-test (*P* < 0.01). **(C)** AlphaScreen assay for *Arabidopsis* FT–FD interaction. AGIA-tagged AtFD was incubated with biotinylated AtFT. Data are represented as mean ± SD of three independent experiments (*n* = 3).

To confirm the presence of protein–protein interactions between LhFT proteins and LhFD, we employed the GAL4 yeast two-hybrid system (Figure 7A). Yeast harboring AD:LhFT1 and BK:LhFD grew on quadruple-dropout medium, which was indicative of protein–protein interactions (Figure 7A). However, yeast cells harboring the reversed vector combination, BK:LhFT1 and AD:LhFD, did not survive on quadruple-dropout medium. *In vitro* protein–protein interaction assay, AlphaScreen, also showed that LhFT1 protein interacted with LhFD (Figure 7B). This signal intensity emitted by LhFT1–LhFD is very similar to that by AtFT–AtFD (Figure 7C). In addition, LhFT6–LhFD and LhFT8–LhFD interactions were also detected with weaker signals than that of LhFT1-LhFD interaction, implying their weak but positive interactions with LhFD (Figure 7B). Taken together, these results suggest that the LhFT1, LhFT6, and LhFT8 proteins form heterodimers with the LhFD protein.

## Discussion

To identify the molecular mechanisms underlying floral initiation in Asiatic hybrid lilies, we identified four *FT*-like genes, *LhFT1*, *LhFT4*, *LhFT6*, and *LhFT8*, in the Asiatic hybrid lily ‘Lollypop’ and characterized their functions.

Based on the deduced amino acid sequences of the *LhFT* genes, *LhFT1* and *LhFT8* were classified into the FT-like IA subgroup (Figure 2). *LhFT1* belonged to same clade as *LlFT* from *L. longiflorum* (Leeggangers et al., 2018), whereas *LhFT8* belonged to another clade containing *LfFT1* from *L.* × *formolongi* (Li et al., 2017) and *LiFTL2* from the Asiatic hybrid lily ‘Connecticut King’ (Leeggangers et al., 2018; Figure 2). A complementation experiment using the *Arabidopsis ft-10* mutant indicated that both *LhFT1* and *LhFT8* are potential inducers of the floral transition in Asiatic hybrid lilies (Figures 6A,B). The floral-inducer activity of *LhFT1* was stronger than that of *LhFT8*. The yeast two-hybrid and AlphaScreen analyses showed that LhFD protein interacted strongly with the LhFT1 protein; however, the interaction between LhFD and LhFT6/8 proteins was not detected (Figures 7A,B). The yeast two-hybrid assay can indirectly detect yeast survival and cannot control the expression levels of a transgene fused with the relative large GAL4 protein. We believe that AlphaScreen is more sensitive than the yeast two-hybrid assay because it detects the direct interaction between native proteins with short tag. Therefore, AlphaScreen would be useful to evaluate FT–FD interactions in plants.

The expression levels of *LhFT1* increased gradually in bulb scales during chill exposure, followed by a sharp increase at 1 week after planting (Figure 3A). This expression profile of *LhFT1* correlated well with that of a floral identity gene (*LhMADS5*) and with the initiation of floral differentiation (Figures 1, 3). *L.* × *formolongi* is a lily that blooms within 1 year after sowing and expresses *LfFT* in its leaves (Li et al., 2017). *TgFT2* from *T. gesneriana* is expressed in the stem and leaves during rapid shoot elongation and in flowers in the blooming period (Leeggangers et al., 2018). However, previous studies did not investigate the expression of *FT*-like genes in bulb scales of these lilies. Perhaps *LhFTl* ortholog might be upregulated in the bulb scales of other species belonging to the family Liliaceae in response to environmental changes. In many plant species, *FT*-like genes are expressed in leaves under flowering-inducing conditions, followed by transportation via the phloem to the SAM (Abe et al., 2005; Corbesier et al., 2007; Tamaki et al., 2007). However, the four *LhFT* genes identified here in an Asiatic hybrid lily were expressed mainly in bulb scales, in which the expression levels were higher than those detected in leaves (Figure 5). *L. leichtlinii* ‘Hakugin’ also led to the detection of a strong expression of *LhFT1* in their bulb scales (Supplementary Figure 4). In ‘Hakugin,’ which underwent flower initiation 6 weeks later than ‘Lollypop,’ the expression of *LhFT1* also induced at 4 weeks after planting (Supplementary Figure 4). The later flowering of ‘Hakugin’ than ‘Lollypop’ correlated well with the delayed expression initiation of *LhFT1* in its scales (Figure 3A and Supplementary Figure 4). These results strongly suggest that *LhFT1* is a floral inducer in Asiatic hybrid lilies. Scales are leaf-like organs that make up the bulb, in addition to the true photosynthetic leaves and inflorescence base (Rees, 1972). The investigation of the levels of *NFT1* in Chinese narcissuses revealed its expression in apices of bulbs and leaves, but not in scales of bulbs (Li et al., 2013). Thus, this study provided new knowledge, in that the expression of *FT*-like transcripts was induced in bulb scales of an Asiatic hybrid lily.

The deduced amino acid sequence of *LhFT8* was highly similar to that of *LlFT* from *L. longiflorum* (Leeggangers et al., 2018) and orchid *FT* transcripts (Hou and Yang, 2009; Jang et al., 2015). Since cold exposure for 9 weeks upregulates *LlFT* in the meristem of bulbs of *L. longiflorum*, *LlFT* has been proposed to be involved in the vernalization response of lilies. The overexpression of *LlFT* in wild-type *Arabidopsis* induces a mild early-flowering phenotype, and *LlFT*-overexpressing transgenic lilies exhibited flowering under non-inductive condition (Leeggangers et al., 2018). An *LhFT8*-overexpressing *Arabidopsis ft-10* mutant also exhibited a mild early-flowering phenotype (Figure 6B).

Interestingly, *LhFT8* expressed its four alternative mRNA variants in the bulbs of Asiatic hybrid lilies during chilling exposure (Figures 3, 4). The expression of one functional and three alternative *LhFT8* variants was induced in both the scales and SAM of bulbs during the initial 8 weeks of chilling (Figure 3). The functional *LhFT8* mRNA detected in bulb scales and SAMs disappeared 3 weeks and 1 week after planting, respectively. The expression of the functional *LhFT8* gene was downregulated after the induction of floral meristems, unlike that observed for *LhFT1*. *LlFT* was considered to be involved in creating meristem competence to flowering cues in *L. longiflorum* (Leeggangers et al., 2018). Therefore, *LhFT8* is likely to be closely associated with the vernalization response of these plants, rather than act as a floral inducer. In perennial species, *FT*-like genes regulate growth cessation and dormancy (Bohlenius et al., 2006; Hsu et al., 2006, 2011; Ream et al., 2012). In perennial poplar, *PtFT1* expression in winter initiates the transition of vegetative meristems to the reproductive phase, whereas *PtFT2* controls vegetative growth by inducing growth cessation, bud set, and dormancy in the growing season (Hsu et al., 2006, 2011). In biennial sugar beets, the *FT* duplication products *BvFT1* and *BvFT2* have divergent functions (Pin et al., 2010). *BvFT2* is a flowering inducer, whereas *BvFT1*, resulting in part from a three-amino-acid change in segment B of BvFT2, is a flowering repressor, despite being in the FT-like IA subgroup (Pin et al., 2010, 2012). *BvFT1* is expressed at the juvenile stage, whereas *BvFT2* is expressed during the reproductive stage (Pin et al., 2012). The deduced amino acid sequence of *LhFT8* showed 72.2% identity with that of *LhFT1*, and a single residue of segment B of LhFT8 was replaced by Glu (E) at position 132 (Supplementary Figure 2). Therefore, this single residue alteration of segment B between LhFT8 and LhFT1 might be responsible for their functional differentiation. Alternative splicing occurs in >61% of intron-containing genes in *Arabidopsis*, 60% in *Drosophila melanogaster*, and more than 95% in humans (Capovilla et al., 2015). However, the biological significance of most alternative splicing event in plants remains largely unknown. In *Brachypodium distachyon*, which is a model plant for major crop cereals, *BdFT2* undergoes age-dependent alternative splicing, resulting in two splicing variants, *BdFT2α* and *BdFT2β* (Qin et al., 2017). We were not able to clearly demonstrate the molecular mechanism underlying the *LhFT8* alternative splicing. Alternative variants of *LFT6* and *LhFT8* seem to lack segment B in their C-terminal ends (Supplementary Figure 2). Segment B is an important domain for binding to 14-3-3 proteins (Taoka et al., 2011). Because AlphaScreen showed that LhFT6 and LhFT8 also interacted with LhFD (Figure 7), C-terminal deficiency in LhFT6 and LhFT8 proteins might function as either the negative auto-regulators or antagonists of LhFT1 protein.

Upregulation of *LhFT6* in bulb scales after planting was detected prior to the timing of floral initiation (Figures 3A, 5). The overexpression of *LhFT6* did not complement the delay in flowering in *Arabidopsis ft-10* (Figure 6D). The LhFT6 protein showed weaker interaction with LhFD protein than LhFT1 (Figure 7B). *LhFT6* was classified into the FT-like 1B subgroup (Figure 2), which includes *AcFT6* from *A. cepa* (Lee et al., 2013) and *TgFT3* from *T. gesneriana* (Leeggangers et al., 2018). *TgFT3* expression was initiated earlier and increased in the stem and leaves during rapid shoot elongation. *TgFT3* overexpression weakly repressed floral transition in *Arabidopsis*, and *TgFT3* might act as negative regulator of flowering in tulips (Leeggangers et al., 2018). Further studies are required to identify the function of *LhFT6*.

*LhFT4* exhibited a length of 1,164 bp and encoded 112 amino acids, i.e., it was shorter than other FT/TFL proteins (Supplementary Figure 2). The deduced amino acid sequences of *LhFT4* were categorized into the FT-like IB clade (Figure 2), but contained a histidine (H) residue that is known to be important for the specific and unique TFL1 function in *Arabidopsis* (Supplementary Figure 2). The LhFT4 protein showed 98.1% identity with that encoded by *LiFTL3* from the *Lilium* spp. ‘Connecticut King’ (Leeggangers et al., 2018). However, the *LiFTL3* mRNA encoded sequences of 181 amino acids, suggesting the existence of an *LhFT4* allele encoding longer amino acid sequences in other cultivars of Asiatic hybrid lilies. The overexpression of *LhFT4* did not affect the flowering time in either *Arabidopsis ft-10* plants (Figure 6C). In addition, *LhFT4* expression was detected at constitutive levels in all samples (Figures 3, 5). Therefore, we assumed that *LhFT4* does not function in flowering signaling in Asiatic hybrid lilies.

Asiatic hybrid lilies create meristem competence to flowering cues by exposing bulbs to low temperatures, and then the bulbs sprout in spring and flower in late spring to early summer (Okubo and Sochacki, 2012). In the most cultivars, including ‘Lollypop,’ flower bud differentiation starts and is completed after shoot emergence (Ohkawa et al., 1990). *LhFT8* was associated with the vernalization response in lily bulbs and was speculated to control the expression levels of *LhFT8* mRNA by occurring as splicing variants. When the bulb is released from cold exposure, *LhFT1* expression is induced in bulb scales, and floral transition occurs in SAM after shoot emergence. This study provides the first evidence that the expression of *LhFT1* in bulb scales is regulated by alterations of temperature in Asiatic hybrid lilies and contributes to flowering initiation. Furthermore, the generation of *LhFT8* splicing variants in lily bulbs during cold exposure might be involved in winter memory (Bouche et al., 2017). Further studies are required to reveal the function of *LhFT8* alternative variants and their generation mechanism. Our findings can help reveal the molecular mechanism of flowering and vernalization in geophytes.

## Data Availability Statement

The datasets presented in this study can be found in online repositories. The names of the repository/repositories and accession number(s) can be found below: https://www.ddbj.nig.ac.jp/, LC544113; https://www.ddbj.nig.ac.jp/, LC544114; https://www.ddbj.nig.ac.jp/, LC544115; https://www.ddbj.nig.ac.jp/, LC544116; https://www.ddbj.nig.ac.jp/, LC544117; https://www.ddbj.nig.ac.jp/, LC544118; https://www.ddbj.nig.ac.jp/, LC544119; https://www.ddbj.nig.ac.jp/, LC544120; https://www.ddbj.nig.ac.jp/, LC544121.

## Author Contributions

KK, TN, and MY conceived the experiments. KK and JK performed the gene expression analysis. KK and TN performed the yeast two-hybrid analysis and complement experiments of *ft-10*. KN, AN, and TS performed the AlphaScreen analysis. TN, KN, and MY wrote the manuscript. All authors approved the manuscript.

## Conflict of Interest

The authors declare that the research was conducted in the absence of any commercial or financial relationships that could be construed as a potential conflict of interest.
