# Supplementary material for: Expression of LhFT1, the Flowering Inducer of Asiatic Hybrid Lily, in the Bulb Scales
Source: Front Plant Sci. 2020 Nov 9;11:570915. doi: 10.3389/fpls.2020.570915 (PMC7693649; doi:10.3389/fpls.2020.570915)
Supplement: Supplementary file 1 [file Data_Sheet_1.PDF]

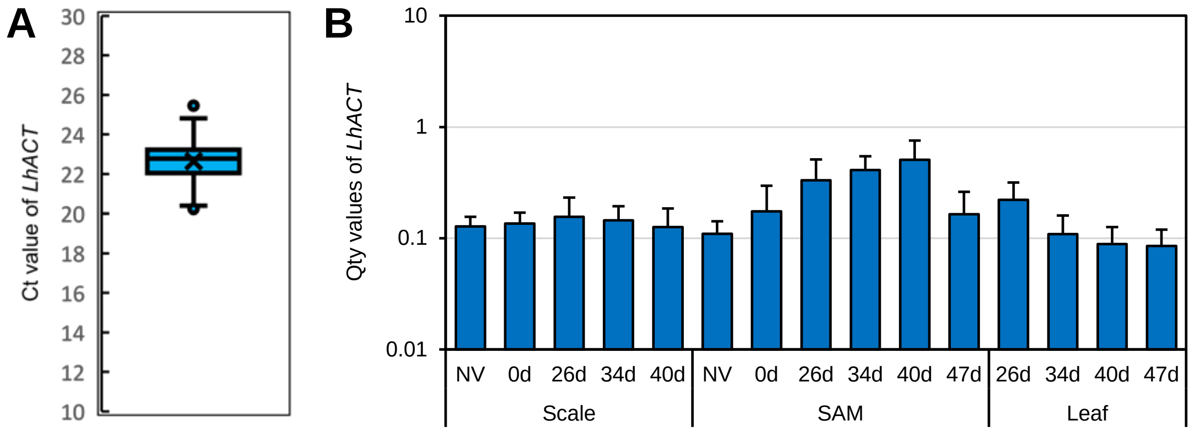

Supplementary Figure. S1. Evaluation of *LhACT* as an internal control in RT-qPCR analysis. A Ct values of *LhACT* in all samples of Asiatic hybrid lily 'Lollypop'. **B** Qty values of *LhACT* among scales, SAMs, and leaves in different development stages. Values indicate mean ± SE.

```
                             DV                                           R        DPDXP        Y/H
LhFT1  -------MENRSDPLAVGRVVGDVLDPFVRRISLRVVYGSKVVNNGREFKPSEVVNQPRVEIGGNDLRNFYTLVMVDPDAPSPSNPNLREYLHWLVT  93
LfFT1  -------MENRSDPLAVGRVVGDVLGPFVRRVSLRVVYGSKVVNNGKEFKPSEVVNQPRVEIGGNDLRNFYTLVMVDPDAPSPSNPNLREYLHWLVT  93
LiFTL2 -------MENNSDPLIVSRVVRDVLDPFTRKISLRVVYGSKVVTNGREFKPSEVVNQPRVEIGGNDLRNFYTLVMVDPDAPSPSNPNLREYLHWLVT  93
TgFT2  -------MENSSDPLAIGRVVGDVIGPFIRRLSIRVVYGSKVVNNGKEFKPSEVVNQPRVDIGGNELRNFYTLVMVDPDAPSPSNPNLREYLHWLVT  93
OsHd3a ----MAGSGRDRDPLVVGRVVGDVLDAFVRSTNLKVTYGSKTVSNGCELKPSMVTHQPRVEVGGNDMRTFYTLVMVDPDAPSPSDPNLREYLHWLVT  96
LhFT8  -----MRRSSGLDPLVTGRVIGDVLDPFISSVNLSVRYQSRGVANGCEFRPSAVVEQPRVEVGGNDLRTVYTLILVDPDAPSPSNPYLREYLHWLVA  95
LhFT8’ -----MRRSSGLDPLVTGRVIGDVLDPFISSVNLSVRYQSRGVANGCEFRPSAVVEQPRVEVGGNDLRTVYTLILVDPDAPSPSNPYLREYLHWTEG
LlFT   ---MNMRRSSGLDPLVTGRVIGDVLDPFISSVNLSVRYQSRGVANSCEFRPSAVVEQPRVDVGGNDLRTVYTLILVDPDAPSPNNPYLREYLHWLVS  97
AtFT   ------MSINIRDPLIVSRVVGDVLDPFNRSITLKVTYGQREVTNGLDLRPSQVQNKPRVEIGGEDLRNFYTLVMVDPDVPSPSNPHLREYLHWLVT  94
LhFT6  ---------MSRDPLVLGEVVGDVLDQFIKSATMRVVY-EKELTNGTRLKQSAVVNAPLIEIGGRDVRTLYTLVMVDADAPSPSNPTQREYLHWLVT  90
LhFT6’ ---------MSRDPLVLGEVVGDVLDQFVKSATMRVVY-EKELTNGTRLKQSAVANAPLIEIGGRDVRTLYTLVMVDADAPSPSNPTQREYLHWLVT
AcFT6  MQVKMLRERVARDPLVLGQIIGDVVDPFTKSVNLKVVYGDKEVSNGTRLRQSMVINQPRVTIEGRDSRTLYSLVMINPDAPSPTNPTHREYLHWLVT 100
TgFT3  ---------MSRNPLVLNEVVGDVLDPFVKSATMRVVY-DKEITNGTRLSQLAVADEPRVEIGGHDVRTLYTFVMVDADAPSPSTPSQREYLHWLVT  90
TgFT1  -------MSRERDPLVVGRVVGDVLDPFTRTTTLKVTFGTREVTNGRELKPSHVVNQPRVEIGGDDLGSFYTLLMVDPDAPNPSDPHLREYLHWLVT  93
LiFTL1 -------MSRERNPLVVGRVIGDLLDPFIRTIPIRVTYGTREVTNGRELKPSHVVNQPKVEIGGDDLRTFYTLVMVDPDAPNPSEPILREYLHWMVA  93
LhFT4  --------------------------------------------------------------------------MVDPDAPSPSNPTEREHLHWMVT  23
LiFTL3 ---MFSSSGQSREPLVVGRVVGDILDPFVRTATLKVVYNNRELTNGSELKPSMVASEPRLEIGSRDMRTLYTLVMVDPDAPSPSNPTEREHLHWMVT  97
AtTFL1 ---MENMGTRVIEPLIMGRVVGDVLDFFTPTTKMNVSYNKKQVSNGHELFPSSVSSKPRVEIHGGDLRSFFTLVMIDPDVPGPSDPFLKEHLHWIVT  97
                    **       *    *       *        *        *   *                    * * * *  *     ***

                                              R          Q
LhFT1  DIPGGTAASFGQEKTCYESPYPRAGIHRFILVLFQQ-LGRQTVYAPD-WRQNFNTRDFAEHYNLGSPVAAVYFNCQRESGSGGRRMFA--- 176
LfFT1  DIPGGTAASFGQVKTCYESPYPRAGIHRFVLVLFQQ-LGRQTVYASD-WRQNFNTRDFAEHYNLGSPVAAVYFNCQRESGSGGRRMFA--- 176
LiFTL2 DIPGGTAASFGQEKTCYESPYPRAGIHRFVLVLFQQ-LGRQTVYAPD-WRQNFNTRDFAEHYNLGSPVAAIYFNCQRESGSGGRRMYA--- 176
TgFT2  DIPGSTAVNYGQEQTCYESPQPRAGIHRFVFVLFQQ-LGRQTVYAPG-WRQNFSTRDFAELYNLGPPVAAVYFNCQREAGSGGRRMYP--- 176
OsHd3a DIPGTTAASFGQEVMCYESPRPTMGIHRLVFVLFQQ-LGRQTVYAPG-WRQNFNTKDFAELYNLGSPVAAVYFNCQREAGSGGRRVYP--- 179
LhFT8  DIPGTTGVTFGHEIVRYECPRPTLGIHRFVFVLFQQ-LGRETVYAPG-WRQNFNTRNFSDLYNLGSPVATVYFNCQRESGSGGRRLPTT-- 179
LhFT8’ SMFLQ-------------------------------------------------------------------------------------- 100
LlFT   DIPGTTGVTFGHEIVRYECPRPSLGIHRFVFVLFQQ-LGRGTVYAPG-WRQNFNTRNFSDLYNLGSPVATVYFNCQRESGSGGRRLPTT-  181
AtFT   DIPATTGTTFGNEIVCYENPSPTAGIHRVVFILFRQ-LGRQTVYAPG-WRQNFNTREFAEIYNLGLPVAAVFYNCQRESGCGGRRLRT--- 177
LhFT6  DIPEGRDARFGNEIVSYEAPRASTGIHRVVFTLFRQ-EVQQTVYAPG-WRPNFNTRDFASYYNLGSPVAAIYFNCIREQGCGGRRF----- 171
LhFT6’ DIPEGRDARFGGSHQYYHLL------------------------------------------------------------------------ 110
AcFT6  DIPETVDASYGNEIVQYESPWTPTGIHRIVFVLFKQ-QIQQTVYAPG-WRQNFYTRDFAAYYNLGSPVAAVYFNCHRESGCGGRRFSGLC- 185
TgFT3  DIPEGRDARYGNEIVSYEGPRAPTGIHRVVFALFRQ-EVQQTVYAPG-WRPNFNTRDFASYYNLGDPVAAIYFNCQREQGCGGRRF----- 171
TgFT1  DIPGTTGPNYGQELVSYESPRPTSGIHRFVFILFKQHLGRQSVYAPG-WRQNFNTRDFAEIYNIGPPVAAVYFNGQRESGSGGRRLYP--- 177
LiFTL1 DIPGTTGPDYGHELVSYESPRPTSGIHRFVFVLVKQHMGRQSVYAPG-WRQQFNTRDFAEIYNLGPPVAAVYFNGQRESGSGGRRMYH--- 177
LhFT4  DIPETAGVRFGNEIVSYESPRPKAGIHRFVFVLFRQ-TVQQTIYAPG-WRQNFNTRDFSALYNLGPPVASIYFNCQRAGGCGGRRYQPVSG 112
LiFTL3 DIPETTGVRFGNEIVSYESPRPKAGIHRFVFVLFRQ-TVQQTIYAPG-WRQNFNTRDFAALYNLGPPVASIYFNCQRAGGCGGRRYPNQ-- 181
AtTFL1 NIPGTTDATFGKEVVSYELPRPSIGIHRFVFVLFRQ-KQRRVIFPNIPSRDHFNTRKFAVEYDLGLPVAAVFFNAQRETA----------- 173
        **       *     ** *    **** *  *  *             *  * *  *   *  *  *** *           ****
                                        Q/E
                                         Segment B
```

Supplementary Figure S2. Amino acid alignment of selected monocot *FT*-like sequences. Sequences were aligned using ClustalW. The accession numbers of each FT-like protein are shown in Fig. 2. LhFT6' and LhFT8' was the deduced amino acid sequences of alternative *LhFT6* and *LhFT8* mRNA, respectively. The highlights indicate amino acid residues that are critical as flowering activators and repressors. DV and R have been known or suspected to be involved in the transport and binding surface of 14-3-3 protein. DPDXP is conserved among PEBP family proteins. Y/H is involved in the functionalization of floral activator and repressors. Segment B is important for the binding to 14-3-3 protein.

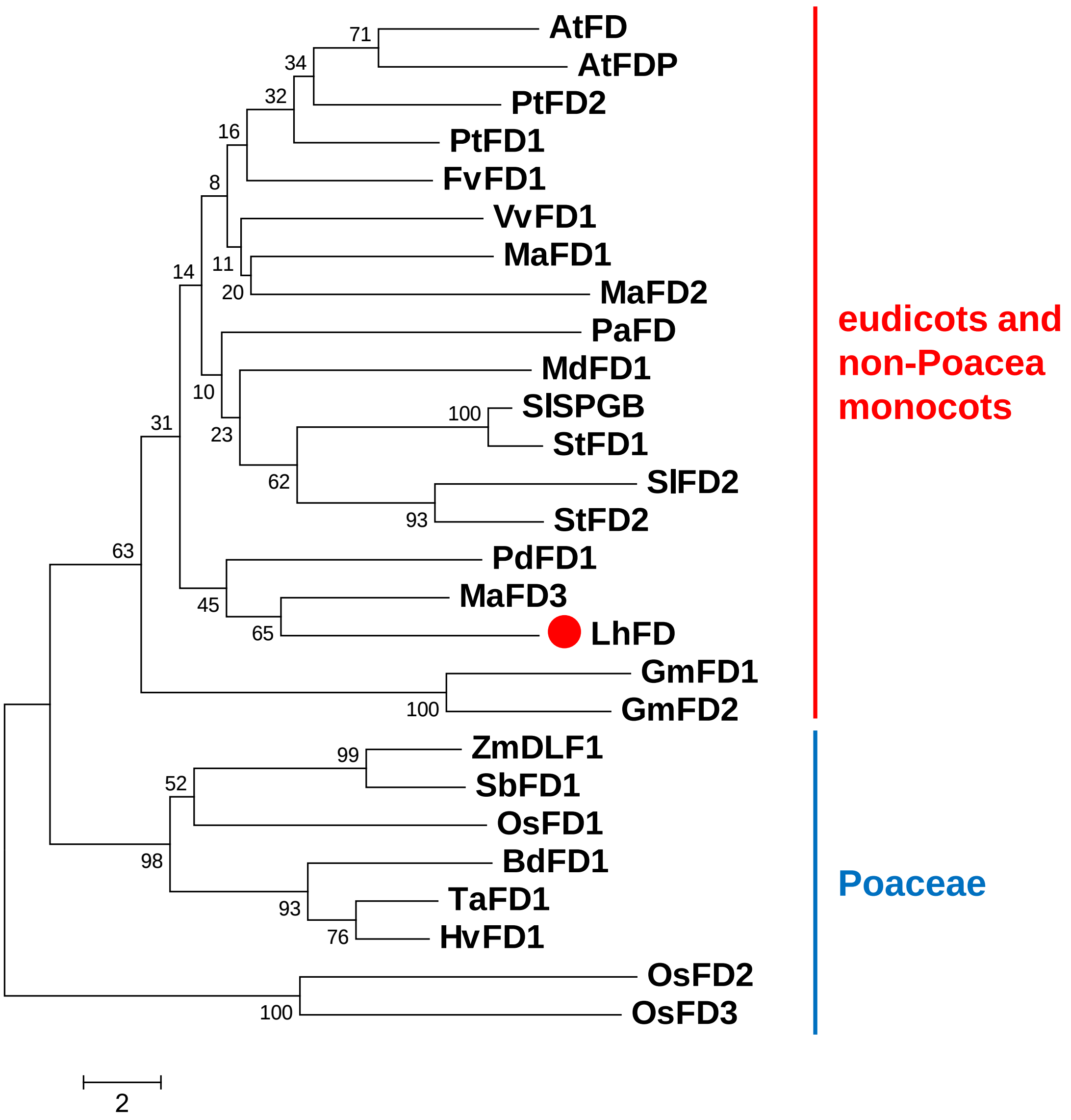

Supplementary Figure S3. Phylogenetic tree of FD-like protein sequences from different monocot plants. The phylogenetic tree was constructed using the neighbor-joining method. Bootstrap values from 1,000 replicates were used to assess the robustness of the tree. The scale indicates the average number of substitutions per site. The *FD*-like gene names and GenBank accession numbers were as follows: *Arabidopsis thaliana AtFD* (At4g35900) and *AtFDP* (At2g17770); *Brachypodium distachyon BdFD1* (Bradi4g36587); *Fragaria vesca FvFD1* (mrna14556.1-v1.0-hybrid); *Glycine max GmFD1* (Glyma04g02420) and *GmFD2* (Glyma06g02470); *Hordeum vulgare HvFD1* (BAK04622); *Lilium* hybrid *LhFD* (this study); *Malus* × *domestica MdFD2* (MDP0000636541); *Musa acuminate MaFD1* (GSMUA_Achr1T02630_001), *MaFD2* (GSMUA_Achr5T11470_001), and *MaFD3* (GSMUA_Achr9G24090_001); *Oryza sativa OsFD1* (Os09g0540800), *OsFD2* (Os06g0720900), and *OsFD3* (Os02g0833600); *Phalaenopsis aphrodite PaFD* (KJ609180); *Phoenix dactylifera PdFD1* (PDK_30s1175071g003); *Populus trichocarpa PtFD1* (POPTR_0005s11140.1) and *PtFD2* (POPTR_0005s26480); *Solanum lycopersicum SlSPGB* (Solyc02g083520.2.1) and *SlFD2* (Solyc02g061990.2.1); *S. tuberosum StFD1* (Sotub02g026810.1.1) and *StFD2* (Sotub02g009830.1.1); *Sorghum bicolor SbFD1* (Sb02g031340); *Triticum aestivum TaFD1* (CK206464); *Vitis vinifera VvFD1* (GSVIVT01009970001); *Zea mays DLF1* (GRMZM2G067921).

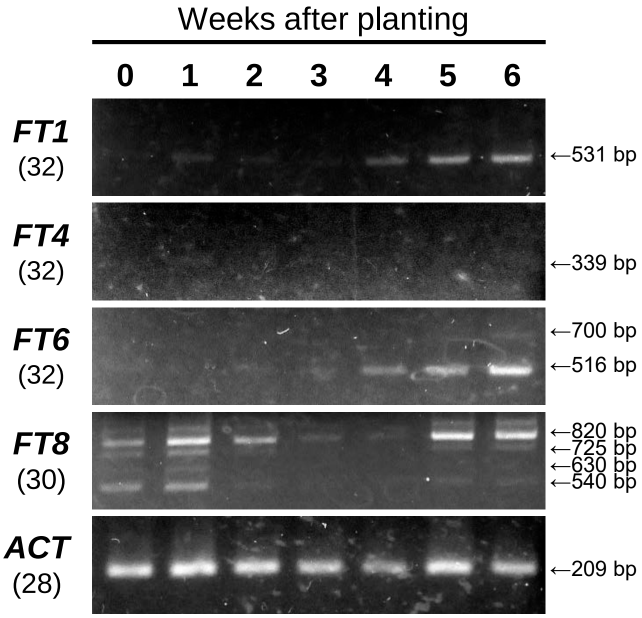

Supplementary Figure S4. Expression patterns of *LhFT* and flowering-related genes in *L. leichtlinii* 'Hakugin' bulbs after planting. Semiquantitative RT-PCR analysis of *LhFT1, LhFT4, LhFT6, LhFT8, LhFD, LhMADS5*, and *LhACT* genes using scales. Scales were collected from bulbs grown for 0, 2, 3, 4, 5, and 6 weeks after planting. The gene names and number of cycles are indicated to the left of the panel. The amplified fragment lengths are indicated to the right of the panels. The 518-bp fragment of *LhFT6* corresponds to the normal transcript, whereas the 700-bp fragment corresponds to the alternative transcript. The 540-bp fragment of *LhFT8* corresponds to the normal transcript, whereas the 630-bp, 725-bp, and 820-bp fragments correspond to the alternative transcripts.

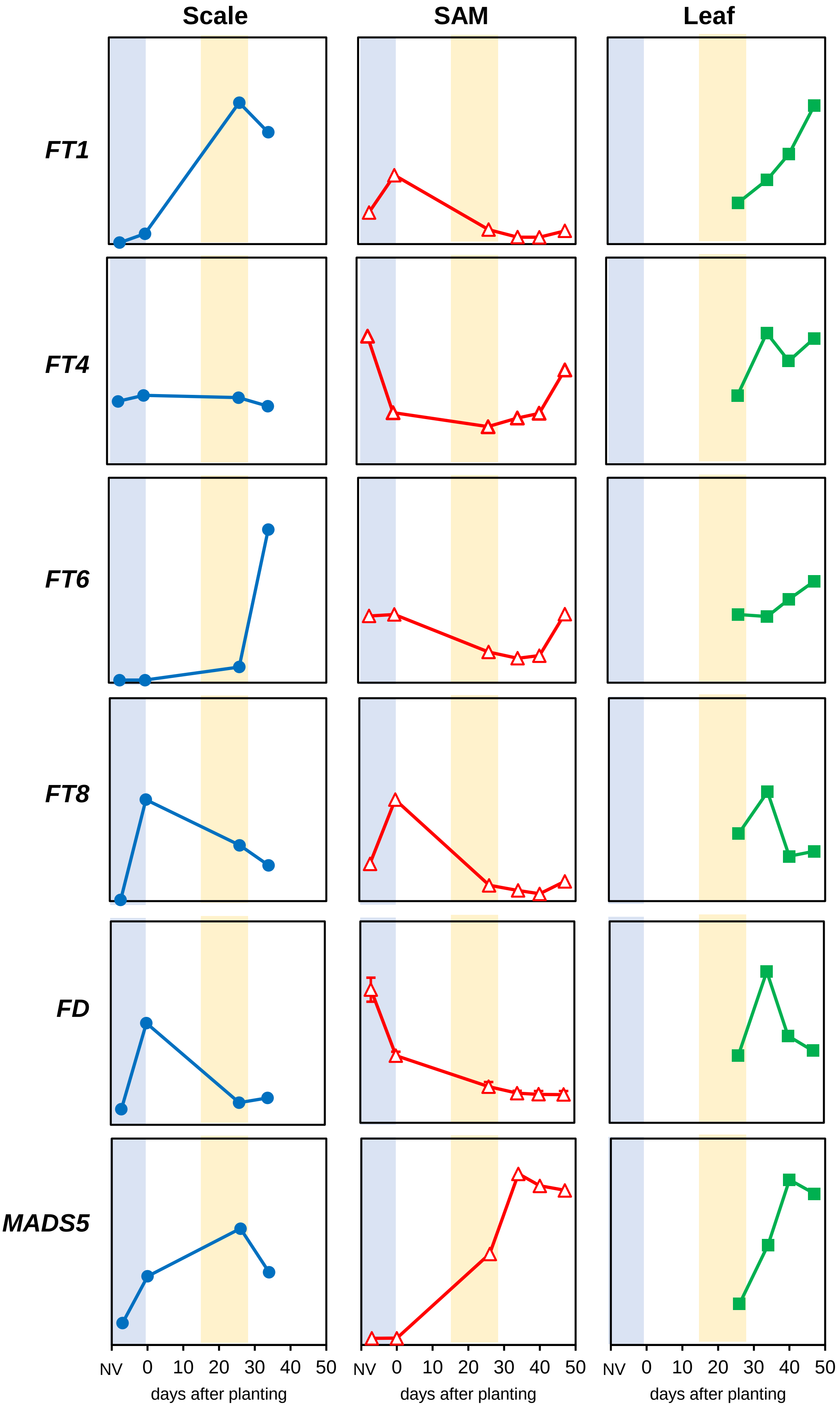

Supplementary Figure S5. The expression profiles of *LhFT* and flowering-related genes in scales, SAM and leaves of 'Lollypop'. The relative expression of scales, SAM in Fig. 4 was converted as the average expression levels at 0 day after planting is 1. The relative expression of leaf is converted as the average expression levels at 24 days after planting is 1. Blue and orange fill boxes indicated chilling exposure period for 4 months and floral initiation period, respectively.
